# Neutrophil Gelatinase-Associated Lipocalin for the Differentiation of Mucinous Pancreatic Cystic Lesions

**DOI:** 10.3390/ijms25063224

**Published:** 2024-03-12

**Authors:** Miruna Patricia Olar, Maria Iacobescu, Sorana D. Bolboacă, Cristina Pojoga, Ofelia Moșteanu, Radu Seicean, Ioana Rusu, Oana Banc, Cristina Adela Iuga, Andrada Seicean

**Affiliations:** 1 Department of Gastroenterology, “Iuliu Hațieganu” University of Medicine and Pharmacy, Victor Babeș Str., no. 8, 400012 Cluj-Napoca, Romania; miruna.olar@gmail.com (M.P.O.); cristina.pojoga@ubbcluj.ro (C.P.); ofeliamosteanu@gmail.com (O.M.); rusuioana@elearn.umfcluj.ro (I.R.); andradaseicean@gmail.com (A.S.); 2 Research Center for Advanced Medicine MedFUTURE, “Iuliu Hațieganu” University of Medicine and Pharmacy, Louis Pasteur Str., nr. 4-6, 400349 Cluj-Napoca, Romania; maria.iacobescu@medfuture.ro (M.I.); iugac@umfcluj.ro (C.A.I.); 3 Department of Medical Informatics and Biostatistics, “Iuliu Hațieganu” University of Medicine and Pharmacy, Louis Pasteur Str., no. 6, 400349 Cluj-Napoca, Romania; 4 Regional Institute of Gastroenterology and Hepatology, Croitorilor Str., no. 19-21, 400162 Cluj-Napoca, Romania; banc.oana@umfluj.ro; 5 International Institute for Advanced Study of Psychotherapy and Applied Mental Health, Department of Clinical Psychology and Psychotherapy, Babeș-Bolyai University, Sindicatelor Str., no. 7, 400029 Cluj-Napoca, Romania; 6 First Department of Surgery, “Iuliu Hațieganu” University of Medicine and Pharmacy, Clinicilor Str., no. 3-5, 400006 Cluj-Napoca, Romania; rseicean@yahoo.com; 7 Drug Analysis, Department Pharmacy 3, Faculty of Pharmacy, “Iuliu Hațieganu” University of Medicine and Pharmacy, Louis Pasteur Str., no. 6, 400349 Cluj-Napoca, Romania

**Keywords:** cystic pancreatic lesions, endoscopic ultrasound, neutrophil gelatinase-associated lipocalin (Ngal), pancreas, pseudocyst

## Abstract

Undetermined pancreatic cystic lesion (PCL) differentiation benefits from endoscopic ultrasound (EUS) based on morphology and cyst fluid analysis, but room for new biomarkers exists. Our aim was to assess the intracystic and serum diagnostic value of neutrophil gelatinase-associated lipocalin (Ngal) and interleukin 1 beta (IL-1β) for differentiation of PCLs. This prospective study included patients from one tertiary hospital, evaluated between April 2018 and May 2020. EUS fine-needle aspiration or pancreatic pseudocysts drainage was the source of PCL intracystic liquid. The final diagnosis was based on surgery or EUS results (morphology, cytology, glucose, and CEA—carcinoembryogenic antigen). The intracystic samples were tested for Ngal, IL-1β, glucose, and CEA, and serum for Ngal and IL-1β. We evaluated 63 cysts, 33 pseudocysts, and 30 non-inflammatory cysts. The diagnostic sensitivity and specificity for mucinous PCL was 70.8% and 92.3% for intracystic Ngal (cut-off: 500–800 ng/dL), without correlation with serum Ngal, no matter the inclusion of infected pseudocysts. After exclusion of infected pseudocysts, the sensitivity and specificity for glucose were 87% and 75%, respectively, and for CEA, they were 87.1%, and 96.8%, respectively. Intracystic Ngal shows promise in differentiating mucinous PCLs, but researchers need to conduct further studies to confirm its effectiveness. Intracystic IL-1β and serum Ngal made no diagnostic contribution.

## 1. Introduction

Advanced transabdominal imaging techniques in asymptomatic patients find about 8% of pancreatic cysts, with 4.3% of mucinous lesions [1], and their number and size increase with age [2]. Most pancreatic cystic lesions (PCLs) are pseudocysts, while pancreatic cystic neoplasms represent only 10–15% of lesions [3].

Mucinous PCLs need special attention and follow-up because of their malignant potential, while non-mucinous cystic pancreatic lesions require no further surveillance. Correct preoperative diagnosis is possible in about 72% of cases [4], and the differentiation between pseudocysts and mucinous cystic lesions is difficult, especially in cases of well delineated homogenous lesions in patients with a history of acute pancreatitis [5]. The standard endoscopic ultrasound (EUS) diagnostic based on morphologic features has a sensitivity of 62% and a specificity of 66% [6]. EUS fine-needle aspiration (EUS-FNA) was considered as a promising tool for improving diagnosis, but the sensitivity for diagnosing left-sided pancreatic cysts rose to only 75% [7]. Currently, the diagnosis of mucinous PCL takes into account intracystic fluid assessment of cytology, carcinoembryonic antigen (CEA), glucose, Kirsten rat sarcoma viral oncogene homolog (KRAS), and guanine nucleotide-binding protein alpha-stimulating activity polypeptide (GNAS) assessment with a sensitivity of 46%, 68%, 92%, 55% and 39%, respectively, and a specificity of 89%, 83%, 65%, 93% and 99%, respectively [6]. Also, the combination of CEA (≥20 ng/mL) and glucose level (≤50 mg/dL) showed a sensitivity of 97% and a specificity of 50% [8,9].

Due to the limited value of the above biomarkers, other biomarkers from cystic fluid would be helpful for a correct diagnosis of pancreatic cysts in differentiating inflammatory from neoplastic lesions.

Human neutrophil gelatinase-associated lipocalin (Ngal), or lipocalin 2, is a protein of the lipocalin family; it is a protease-resistant, acute-phase extracellular protein, secreted by neutrophils, which increases in plasma in inflammatory diseases, acute renal injuries, and malignancies [10,11,12].

Ngal has a multifaced role with both pro- and anti-neoplastic effects. It promotes intracellular iron capture and the formation of complexes with matrix metalloproteinase 9 (MMP9) with pro-malignant potential in breast, esophagus, stomach, brain, and thyroid cancer. Ngal also demonstrates anti-neoplastic and anti-metastatic action by inhibiting cell proliferation and invasion. It reduces the phospforylation of focal adhesion kinase (FAK), hypoxia-inducible factor 1—alpha (HIF1A factor), and the synthesis of vascular endothelial growth factor (VEGF) in colon, ovary, and pancreatic cancer [13].

In their systematic review, Roli et al. [14] reported a high heterogeneity of NGAL in pancreatic cancer with a pooled AUC of 0.84, a pooled sensitivity (Se) of 61% (I^2^ = 90.6%), and a pooled specificity (Sp) of 77% (I^2^ = 66.6%).

Healthy people have low serum concentrations of Ngal because of glomerular filtration and reabsorption in the proximal tubes of circulating Ngal. The urinary levels of Ngal are extremely low, but become elevated in case of acute kidney injury because of renal production and reduced reabsorption. An elevated urinary level is an early predictor of renal dysfunction, which is associated with severe acute pancreatitis, higher mortality, and the need for intensive care unit admission. Therefore, the concentration of urinary Ngal is a prognostic factor of severe acute pancreatitis in early stages of the disease [15].

The Ngal gene was overexpressed in the tissue of thirteen cases with pancreatic adenocarcinoma, representing a potential stromal target during treatment [16]. Ngal was higher in the pancreatic juice of chronic pancreatitis and pancreatic cancer patients compared to the normal pancreas (patients without pancreatic disease), but it has not been proved to differentiate between chronic pancreatitis and pancreatic cancer [17].

Previously, pancreatic juice Ngal and serum Ngal had a significant positive inter-correlation with pancreatic juice interleukin (IL) levels [17]. The analysis of the combination of the levels of serum CA 19-9, pancreatic juice Ngal, and IL-8 was helpful in diagnosing adenocarcinoma patients [18]. However, only one study has been carried out on a very small number of patients with PCL, and information about the diagnostic value was not available [19]. Interleukin-1β (IL-1β) [20,21] is a cytokine involved in the inflammatory response, a response that induces Ngal expression in beta cells, which could be a biomarker and potential therapeutic target [22].

Our study aimed to assess the diagnostic value of intracystic and serum Ngal and IL-1β for the detection of mucinous pancreatic cystic lesions.

## 2. Results

**Figure 1:**
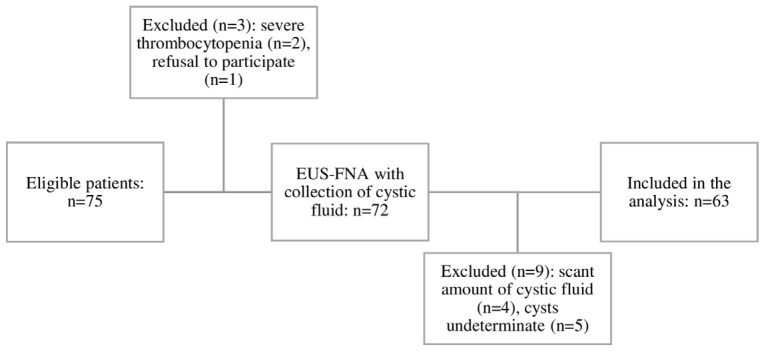
Flowchart from eligibility to inclusion in the study.

Seventy-three subjects with pancreatic cysts were referred for EUS-FNA during the study period, and sixty-three patients were included in the analysis (Figure 1).

### 2.1. Patient Characteristics

Sixty-three patients, aged 18 to 83 years, were evaluated, with 30 pancreatic cystic lesions (47.6%) in group A and 33 pseudocysts (52.3%) in group B (Table 1). The final diagnosis was based on surgery in 8 patients (12.6%), on EUS-FNA cytology in 6 of the malignant cysts with contraindication for surgery (arterial invasion or comorbidities), or on the combination of clinical history, transabdominal imaging and EUS features, and intracystic fluid analysis in 49 patients. Non-inflammatory PCLs were more common in males and had smaller sizes, but there were no differences in localization or patient age (Table 1).

All patients were assessed by CT scan, and 20 (31.7%) by MRI as well, at the time of inclusion. The mean follow-up was 18 months, and six patients died of causes unrelated to the pancreatic cysts.

The mucinous PCLs (n = 24) compared to the non-mucinous PCLs (n = 39) were similar regarding age, sex, and localization, but had a smaller diameter (27 mm vs. 50 mm, *p* = 0.0001) and lower self-reported alcohol consumption (13% vs 36.3%) and smoking behavior (30% vs. 42.4%). The main indication of pseudocyst drainage was abdominal pain (22 patients), vomiting due to gastric compressions (5 patients), and fever (6 patients). Based on intracystic neutrophile levels and/or positive bacterial culture, 8 cases (24%) had an infected pseudocyst.

Patients with suprainfected pseudocysts had significantly lower glucose levels (median = 22 [20 to 25.3], n = 8) compared to non-infected pseudocysts (median = 100 [72 to 144], n = 25) (Mann–Whitney test: *p*-value = 0.00009).

### 2.2. Biomarkers Analysis

Cyst fluid Ngal levels proved significantly higher in patients with pseudocysts than in those with a cystic neoplasm, but serum levels did not show any differences (Table 2). The level of cystic Ngal was higher in the mucinous lesions than in the SCN (median and IQR 729 ng/dL [521 to 769], n = 24 vs. 388 ng/dL [216 to 450], n = 6, *p* = 0.0088—Mann–Whitney test), while patients with a cystic neoplasm exhibited significantly higher values of CEA than those with pseudocysts (*p* = 0.001) (Table 2). No significant differences between different cysts were observed for levels of IL-1β (Table 2).

### 2.3. Evaluated Markers as Diagnostic Tools

**Figure 2:**
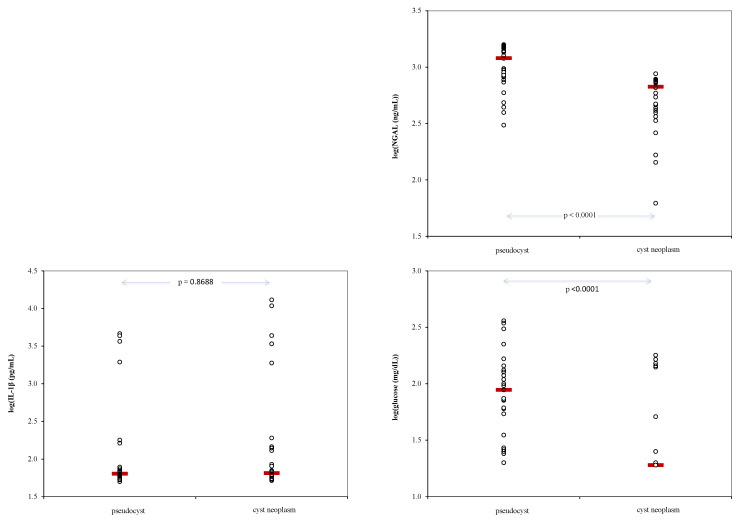
Variation in cyst fluid biomarkers Ngal (neutrophil gelatinase-associated lipocalin), IL-1β, and glucose (logarithmic scale) in inflammatory and non-inflammatory cysts. The dots represent individual data and the red line indicates the value of median.

Cyst levels of Ngal, IL-1β and glucose showed statistically significant differences between patients with pseudocysts compared to those with cyst neoplasm (Figure 2).

**Figure 3:**
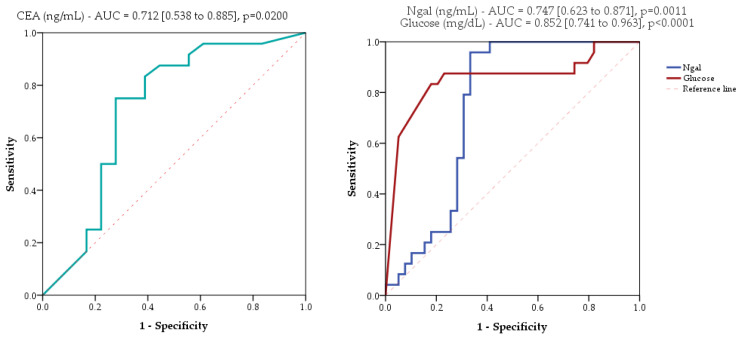
ROC analysis of areas under the curve (AUC) for CEA, Ngal (neutrophil gelatinase-associated lipocalin), and glucose as potential biomarkers. The values in squared brackets are the upper and lower bounds of the confidence interval. Higher values of CEA and lower values of Ngal or glucose indicate malignancy.

Receiver operating characteristic curve (ROC) analysis of mucinous cystic pancreatic lesions showed limited performances of CEA, Ngal, and glucose (Figure 3) and insufficient performances of IL-1β (AUC = 0.503, 95%CI [0.350 to 0.655], *p* = 0.9718; AUC = area under the curve).

Ngal sensitivity in diagnosing mucinous cysts was lower than that of glucose, but the specificity was higher when all the cysts were analyzed. When excluding the patients with infected pseudocysts, sensitivity for Ngal was the same, but the specificity for glucose and CEA was better (Table 3), and the combined glucose + Ngal, CEA + Ngal, CEA + glucose + Ngal testing showed 62.5%, 54.2%, and 50% sensitivity, respectively, and 100% specificity for the diagnosis of mucinous cysts (Table 3).

Histopathological examination was conducted in 8 cases, while conclusive cytology results were obtained from 61 out of 63 patients. The summary data for intracystic Ngal with values between 500 and 800 ng/mL in the identification of malignancy are presented in Table 4. The performances in identification of malignancy when cytology was considered the gold standard diagnostics were as follow: Se = 72.7% (95%CI [54.1–91.3]), Sp = 92.3% (95%CI [83.9–100]), Accuracy = 85.3% (95%CI [76.4–94.2]), +LR = 9.45 (95%CI [3.09–28.89]), −LR = 0.30 (95%CI [0.15–0.59]), +CUI = 0.612 (95%CI [0.395–0.830]), −CUI = 0.791 (95%CI [0.709–0.874]). The results considering cytologic diagnostic indicate the intracyst Ngal biomarker (500–800 ng/mL) as good for screening and fair for case-finding, similar to the results presented in Table 3.

## 3. Discussion

The present work represents the first study assessing the utility of Ngal in cyst fluid and serum for discriminating the undetermined cystic lesions of the pancreas. Ngal is involved in the survival and regeneration of pancreatic cells [10], and our results showed that intracystic levels had a 70% sensitivity for diagnosing mucinous PCLs, but very good specificity (92.3%). The inclusion of infected pseudocysts proved to decrease the specificity for glucose and CEA, compared to 87% and 96.8%, respectively, when these were excluded. Analyzing Ngal as an individual marker proved to be a good tool to screen for pancreatic mucinous cysts, including infected pseudocysts, although these results were not helpful when the concomitant analysis of the three markers was performed.

Discrimination between PCLs remains difficult because the diagnostic sensitivity for mucinous PCLs using EUS remains 71% and 68–88%, respectively, for the two types of lesions when intracystic CEA level is considered [23]. For this reason, intracystic glucose level seems to be promising, as noted in three meta-analyses published in recent years, with 90–92% sensitivity and a specificity of 65–86% [24,25]. Unfortunately, infected pseudocysts may be associated with low glucose levels, making their detection less valuable for ruling out inflammatory cysts [26]. In our study, we had results similar to those published in the literature for the diagnostic role of intracystic glucose, with 87.5% sensitivity and 69.2% specificity. The cut-off level of intracystic CEA of 192 ng/mL for diagnosing mucinous cysts had a sensitivity of 68% [9], close to our results of 75%, which increased to 80% when the cut-off value decreased to 20 ng/mL [8] or to 87% when the cut-off value was 135 ng/mL [8,27].

Mucin-based biomarkers and genetic changes (KRAS and NGAS mutations, telomere fusions, micro RNAs, DNA metilation, long-coding RNAs) have been evaluated for increasing diagnostic accuracy in intracystic liquid, but only KRAS and GNAS have been recognized as having a diagnostic role in mucinous PCLs [6]. In the present study, we studied inflammatory biomarkers such as Ngal and IL-1β. Conversely, Abdellah et al. [28] reported a sensitivity of 84% for diagnosing malignancy in PCLs with a modest specificity of 56% of IL-1β in identifying mucinous cysts. Similar to Kaddah et al. [29], we found no utility for IL-1β in identifying mucinous cysts. 

Previous studies on mice and cell lines proved that Ngal promotes secretion of proinflammatory cytokines in pancreatic stellate cells of the pancreatic adenocarcinoma tumour [30,31]. Ngal in pancreatic juice proved its presence in chronic pancreatitis and pancreatic cancer patients compared to those with normal pancreases [17]. Ngal was found in all 13 EUS fine-needle biopsies in pancreatic adenocarcinoma, influencing the extracellular microenvironment as a target for chemotherapeutic agents [16]. It seems that its precursor is an iron donor to clear-cell renal cell carcinoma, promoting migration and matrix adhesion [32]. Also, it is involved in transporting hydrophobic ligands across cell membranes, modulating immune responses, and promoting epithelial cell differentiation, modulating degradation and activity of metalloprotease 9, which promotes tumor cell invasion and metastasis involved in aggressive types of cancer [33]. In acute pancreatitis, Ngal was a rescue for intracellular reactive oxygen species and a promoter for survival and regeneration of acinar cells, predicting a negative outcome for severe acute pancreatitis [34,35].

However, only one study reported Ngal in PCLs [19], with an Ngal cystic level of 211 ng/dL in the cystic group (which included eleven cystic mucinous neoplasms and 4 SCN). We obtained in our study higher levels, with a median of 728 ng/dL in mucinous group which was significantly different from non-mucinous group. In our study, Ngal value had ranges between 500–800 ng/dL for mucinous PCLs; meanwhile, the mean value for the six cases of SCN was 336 ng/dL and 1116 ng/dL for pseudocysts, resulting in an acceptable sensitivity of 70.8%, no matter of inclusion of infected pseudocysts. Their diagnostic value was inferior to that of glucose and CEA sensitivity value when infected pseudocysts were excluded. However, the combination of these markers proved not to be helpful for diagnosis of mucinous cysts. Also, we could not find any diagnostic value for IL-1β, proving an inflammatory molecular pathway for Ngal different from beta cells in case of PCLs.

The strength of this study consists in its comparative assessments of prospectively collected samples from two groups of non-inflammatory PCLs and pseudocysts referred for drainage, which has not been performed before, and exploration of two different pathways of inflammation. All cystic fluid samples were collected via EUS-FNA, and their manipulation was similar. The measurement of glucose was carried out with a glucometer as part of the current study during the lab assessment for biomarkers and not in a clinical setting, so glucose measurement did not influence the final diagnosis. Also, this is the first time Ngal serum level assessment in conjunction with intracystic level has shwn no value for diagnosis.

Our study has some limitations. First, the evaluated number of patients is limited, and as a consequence, the results must be verified with larger samples before implementation in current practice. Second, surgical specimen confirmation was absent in 22 of 30 patients with PCLs, mainly due to the high number of branch-duct IPMN patients with no need for surgery included. In the absence of a histopathological diagnostic, the reported results reflect strictly the investigated sample and cannot be generalized. Misclassification errors may occur in the absence of the gold standard diagnostic, leading to both false positive and false negative results and overestimation (unnecessary intervention) or underestimation (delay in diagnostic and treatment). Our findings support the evaluation of intracystic Ngal as a diagnostic tool, in the presence of a perfect “gold standard” test and a larger-scale study.

## 4. Materials and Methods

### 4.1. Study Design

This prospective study was conducted between April 2018 and May 2020 at one tertiary medical center, the Regional Institute of Gastroenterology and Hepatology in Cluj-Napoca, Romania, with approval from the institutional review board (3803/29.03.2018). All patients gave written informed consent, in accordance with Helsinki guidelines. The study was registered at clinicaltrials.gov (NCT05568433).

#### 4.1.1. Inclusion Criteria

The patients in group A were diagnosed with undetermined pancreatic cysts identified via computer tomography (CT) and/or magnetic resonance imaging (MRI) and were admitted for EUS-FNA. Group B included patients with symptomatic pseudocysts four weeks from the onset of acute pancreatitis, with abdominal pain, loss of appetite, weight loss, and/or fever, and with an indication of drainage [36].

#### 4.1.2. Exclusion Criteria

Patients were excluded if they (1) refused to participate in or had contraindications for the proposed intervention; (2) had platelet levels <50,000/mm^3^ or INR (international normalized ratio) >1.5; (3) had a solid mass with <50% cystic component; exhibited (4) presence of any of the following: duodenal stenosis, severe chronic pancreatitis, history of pancreatic cancer or major upper abdominal surgery; (5) had congestive heart failure; (6) produced a liquid sample <1 mL; or (7) lacked a final diagnosis at the end of follow-up.

The final diagnostic of the pancreatic cysts was based on surgical specimens or the combination of medical history, EUS morphology, cytology, carcinoembryonic antigen (CEA), and follow-up. Patients with an unclear final diagnosis were excluded.

### 4.2. Procedure

All interventions were performed using a therapeutic linear array echoendoscope (GF-UCT 180 AL5; Olympus, Tokyo, Japan) with an Aloka Prosound F75 ultrasound machine. All interventions were undertaken by the three authors (A.S., O.M, and C.P.), who are experienced in this type of procedure. Patients were under light sedation (intravenous midazolam) or deep sedation (propofol). The patient was positioned in the left lateral decubitus.

The tracking parameters for B-mode EUS were the cyst morphology (the wall, the septae) and the presence or absence of solid components.

After a careful examination in B mode of the entire pancreas, EUS-FNA was performed. Only one passage was performed; first, the fluid was aspirated, and then a sample of the wall or solid component was taken, if present. The patient was observed for one hour and discharged if the procedure was uneventful.

Before the procedure, a blood sample was taken from each patient, centrifuged, and stored at −80 °C freezer until use.

### 4.3. Preparation of Samples

No cytopathologist was present when the samples were collected, and no thorough needle biopsy was performed. The fluid was analyzed macroscopically for color and viscosity. A fluid sample was sent to the laboratory for CEA, amylase, and cytology. In the case of pseudocysts, the fluid sample was sent to the laboratory for culture with antibiogram. A 2 mL sample from the remaining fluid was collected in Eppendorf tubes at the time of the procedure and stored at −80 °C until use (for determination of glucose, Ngal, and IL-1β).

### 4.4. Sandwich Enzyme-Linked Immunosorbent Assays

The levels of Ngal and IL-1β in the cyst fluid and serum were determined using sandwich enzyme-linked immunosorbent assays (ELISA). Samples were individually measured in duplicate following the kits’ instructions (Ngal: Biolegend, Biotechne, Milano, Italy, catalogue number 44,3407, sensitivity = 16.4 pg/mL, intra-assay precision coefficient of variation (CV) = 5.8–7.4%, inter-assay precision CV = 7.0–7.6%, IL-1β: R&D Systems, Minneapolis, MN, United States, catalogue number DLB50, sensitivity = 1 pg/mL, intra-assay precision CV = 2.3–3.4%, inter-assay precision CV = 3.4–7.1%; a calibration curve was generated for each parameter using the protein standard provided by the kit; absorbances were measured with a microplate reader (ClarioStar, BMGLabtech, Ortenberg, Germany), and data acquisition and processing were carried out using the integrated MARS Data Analysis software (v. 3.10 R6 BMG LABTECH, Ortenberg, Germany). A 4-parameter fit calibration curve was used for the quantification, and the final concentration was calculated as the mean of the two measurements.

Carcinoembryogenic antigen (CEA) was determined from the cyst fluid in the clinical laboratory by using ST AIA-PACK CEA (Cat. No. 0025254, TOSOH Corporation, Japan; specifications: sensibility 0.5 ng/mL, specificity 100%, inter-assay precision <2.5%, intra-assay precision <4.6%) following the kit manufacturer’s protocol. Concentrations are reported in ng/mL.

### 4.5. Glucose Level Measurements

Glucose levels were evaluated with a standard glucometer (ONETOUCH Select Plus, LifeScan, Zug, Switzerland, test range 20–600 mg/dL, precision CV =2.07–3.59%, volume of sample required 1 µL). Within 30 min of thawing, each sample was analyzed in duplicate by pipetting 2 µL onto the testing strip [37].

### 4.6. Definitions

The final diagnosis was based on surgical pathology or EUS-FNA cytology or on the correlation of clinical history, CT/MRI, B-mode EUS and CH-EUS morphology, cyst fluid analysis (cytology from the cyst fluid or the solid component, CEA) and follow-up (clinical evaluation, serum CA19-9, abdominal ultrasound every six months and EUS or MRI every year for patients without surgical indication).

Mucinous lesions were denoted as mucinous cystic neoplasms (MCNs), intraductal papillary mucinous neoplasms (IPMNs), or cystic ductal adenocarcinoma. Non-mucinous lesions were considered pseudocysts and serous cystic neoplasms (SCNs). Inflammatory cysts were considered pseudocysts. Non-inflammatory cysts were considered neoplastic cystic neoplasms (IPMNs, MCNs, and SCNs). Undetermined cysts were considered non-operable cysts without a specific diagnosis after transabdominal imaging and EUS-FNA procedures. The cut-off levels for mucinous PCLs were CEA ≥192 ng/mL and glucose ≤ 50 mg/dL [8], and for Ngal, we used the results from the present study.

### 4.7. Statistical Analysis

Quantitative data were reported as mean (standard deviation) when they showed no significant deviations from normal distribution; otherwise, they were reported as median [Q1 to Q3], where Q1 is the 25th percentile and Q3 is the 75th percentile. Quantitative data were reported as numbers and percentages. Comparisons between groups were made with Student’s *t*-test for independent samples or Mann–Whitney test for quantitative data and chi-squared test for qualitative data. Performances of biomarkers in ROC analysis to differentiate mucinous from non-mucinous cysts were reported using sensitivity, specificity, likelihood ratio (positive and negative), accuracy, and clinical utility index (positive and negative).

Statistical analysis was conducted with Statistica program (v.13.5, TIBCO Software Inc., Palo Alto, CA, USA) at a significance level of 5%, two-tailed tests, so the *p*-values less than 0.05 were considered statistically significant.

## 5. Conclusions

Cystic Ngal showed promise in differentiating between certain pancreatic cysts in our study, but further research is needed to confirm its effectiveness. Pancreatic cyst fluid IL-1β and serum Ngal levels were not helpful for this purpose. Future studies should evaluate cystic Ngal in larger populations and alongside a definitive diagnostic test to validate its clinical usefulness.

## Figures and Tables

**Table 1:** Patient demographics and cyst characteristics: inflammatory (pseudocysts) vs. non-inflammatory (cystic neoplasms).

Characteristic	All, n = 63	Pseudocysts, n = 33	Non-Inflammatory PCLs, n = 30	Stat. (*p*-Value)
Age, years ^a^	56 (14)	53.7 (13)	58.6 (14.8)	−1.4 (0.1673)
Sex male ^b^	33 (52.4)	24 (72.7)	9 (30)	11.5 (0.0007)
Alcohol ^b^	17 (27)	13 (39.4)	4 (13.3)	5.4 (0.0199)
Smoker ^b^	22 (34.9)	13 (39.4)	9 (30)	0.6 (0.4347)
Localization ^b^				0.06 (0.9718)
Head	30 (47.6)	16 (48.5)	14 (46.7)	
Body	18 (28.6)	9 (27.3)	9 (30)	
Tail	15 (23.8)	8 (24.2)	7 (23.3)	
Cyst size, mm ^c^	38 [25 to 57.5]	50 [40 to 60]	27 [20 to 35]	4.1 (<0.0001)
Final diagnosis ^b^				n.a.
Cystic ductal adk	2 (3.2)		2 (6.7)	
MCN	3 (4.8)		3 (10)	
BD-IPMN	19 (30.2)		19 (63.3)	
SCN	6 (9.5)		6 (20)	
PC	33 (52.4)	33 (100)		

^a^ Data are reported as mean (standard deviation), Student’s *t*-test for independent samples. ^b^ Data are reported as no. (%), Chi-squared test. ^c^ Data are reported as median [Q1 to Q3], where Q1 is the first quartile and Q3 is the third quartile, Mann–Whitney test. SCN—serous cystic neoplasms, MCN—mucinous cystic neoplasms, BD-IPMN—branch duct intrapapillary mucinous neoplasm, PC—pseudocyst; adk-cystic ductal adenocarcinoma; n.a.—not applicable.

**Table 2:** Markers quantified from cyst fluids and serum in different groups of PCLs (n = 63).

Marker	Pseudocyst, n = 33	Non-Inflammatory PCLs, n = 30	Stat. (*p*-Value)	Mucinous, n = 24	Non-Mucious, n = 39	Stat. (*p*-Value)
Biomarkers in cystic fluid						
NGAL, ng/mL	1199.4 [828.7 to 1510.4]	669.9 [417.2 to 762.7]	5.1 (<0.0001)	728.7 [521.5 to 768.671]	960.9 [538 to 1475.362]	−3.3 (0.0011)
IL-1β, ng/dL	63.8 [58.6 to 74.1]	64.9 [55.9 to 136.4]	−0.2 (0.8688)	62.3 [57.3 to 140.518]	63.8 [58.6 to 79.273]	−0.03 (0.9774)
Glucose, mg/dL	88 [27 to 132]	19 [19 to 23.8]	4.2 (<0.0001)	19 [19 to 20]	88 [26.5 to 138]	−4.7 (<0.0001)
CEA *, n (%)	2 (6.1)	18 (60)	21.1 (<0.001)	18 (75)	2 (5.1)	33.5 (<0.0001)
Biomarkers in serum						
NGAL, ng/mL	648.8 [603.8 to 665.6]	643 [569.7 to 673.1]	0.3 (0.7779)	638.9 [555.3 to 675.4]	651 [608.9 to 666.3]	−0.6 (0.5475)
IL1β, ng/dL	153.1 [143.1 to 160.4]	143.5 [128.5 to 156.3]	−1.35 (0.1774)	142.6 [123.8 to 156.1]	153.1 [143.1 to 160.4]	1.47 (0.141)

PCL—pancreatic cystic lesions; Ngal—neutrophil gelatinase-associated lipocalin; Il-1β—interleukin 1 beta; CEA—carcinoembryonic antigen; * higher than 192 ng/dL; data are expressed as median [Q1 to Q3], where Q is the quartile and compared with Mann–Whitney test, except CEA when the data are expressed as no. (%); comparisons made with chi-squared test.

**Table 3:** Performances of investigated intracystic markers for ruling-in (case finding) and ruling-out (screening) of mucinous cystic pancreatic lesions in all pancreatic cysts and after excluding infected pseudocysts.

	Criterion	Ngal 500–800 ng/mL	Glucose ≤50 mg/dL	CEA ≥192 ng/mL	Ngal 500–800 ng/mL	Glucose ≤50 mg/dL	CEA ≥192 ng/mL
Characteristic							
No. of pts		All cysts (n = 63)			Cysts without infected pseudocysts * (n = 55)		
Contingency							
True positive		17	21	21	17	21	18
False positive		3	12	5	2	4	1
False negative		7	3	3	7	3	6
True negative		36	27	34	29	27	30
χ^2^ (*p*-value)		27.3 (<0.0001)	19.2 (<0.0001)	34.2 (<0.0001)	24.8 (<0.0001)	30.4 (<0.0001)	30.8 (<0.0001)
Sensitivity, %		70.8 [52.6 to 89.0]	87.5 [74.3 to 100]	87.5 [74.3 to 100]	70.8 [52.6 to 89.0]	87.5 [74.3 to 100]	75.0 [57.7 to 92.3]
Specificity, %		92.3 [83.9 to 100]	69.2 [54.7 to 83.7]	87.2 [76.7 to 97.7]	93.5 [84.9 to 100]	87.1 [75.3 to 98.9]	96.8 [90.6 to 100]
+LR		9.21 [3.01 to 28.14]	2.84 [1.73 to 4.66]	6.83 [2.97 to 15.7]	10.98 [2.80 to 42.98]	6.78 [2.68 to 14.14]	23.25 [3.3 to 162.1]
−LR		0.32 [0.17 to 0.59]	0.18 [0.06 to 0.53]	0.14 [0.05 to 0.42]	0.31 [0.17 to 0.59]	0.14 [0.05 to 0.42]	0.26 [0.13 to 0.52]
Accuracy, %		84.1 [75.1 to 93.2]	76.2 [65.7 to 86.7]	87.3 [79.1 to 95.5]	83.6 [73.9 to 93.4]	87.3 [78.5 to 96.1]	87.3 [78.5 to 96.1]
+CUI		0.602 [0.390–0.814]	0.557 [0.364 to 0.750]	0.707 [0.531 to 0.882]	0.634 [0.427 to 0.840]	0.735 [0.566 to 0.905]	0.711 [0.525 to 0.896]
−CUI		0.773 [0.688 to 0.858]	0.623 [0.503 to 0.743]	0.801 [0.716 to 0.886]	0.754 [0.657 to 0.850]	0.786 [0.686 to 0.882]	0.806 [0.720 to 0.893]
For case-finding		Fair	Fair	Good	Fair	Good	Good
For screening		Good	Fair	Good	Good	Good	Good

Ngal—neutrophil gelatinase-associated lipocalin; CEA—carcinoembryonic antigen; LR—likelihood ratio; CUI—clinical utility index. The performance metrics are reported as point estimator and associated 95% confidence interval defined as [lower bound to upper bound]; * excluding infected pseudocysts.

**Table 4:** Performances of intracystic Ngal (values between 500 and 800 ng/mL) in histopathology and cytology diagnostic in identification of malignancy.

	Histopathology, n = 8	Cytology, n = 61
True positive	6	16
False positive	0	3
False negative	2	6
True negative	0	36
χ^2^ (*p*-value)	n.a.	59.4 (<0.0001)

χ^2^ = chi-squared statistics; n.a. = not applicable.

## Data Availability

Raw data and study materials that support the findings will be available to other researchers from the first author (M.P.O.) upon request.
